# A Novel Diagnostic Feature for a Wind Turbine Imbalance Under Variable Speed Conditions

**DOI:** 10.3390/s24217073

**Published:** 2024-11-02

**Authors:** Amir R. Askari, Len Gelman, Russell King, Daryl Hickey, Andrew D. Ball

**Affiliations:** 1 Department of Engineering, School of Computing and Engineering, The University of Huddersfield, Huddersfield HD1 3DH, UK; a.askari@hud.ac.uk (A.R.A.); a.ball@hud.ac.uk (A.D.B.); 2 Department of Mechanical Engineering, Hakim Sabzevari University, Sabzevar 9617976487, Iran; 3 Sensonics Ltd., 3 Northbridge Rd, Berkhamsted HP4 1EF, UK; russell.king@sensonics.co.uk; 4 Natural Power, 120 Bath St, Glasgow G2 2EN, UK; darylh@naturalpower.com

**Keywords:** signal processing, fault diagnosis, rotating machinery

## Abstract

Dependency between the conventional imbalance diagnostic feature and the shaft rotational speed makes imbalance diagnosis challenging for variable-speed machines. This paper focuses on an investigation of this dependency and on a proposal for a novel imbalance diagnostic feature and a novel simplified version for this feature, which are independent of shaft rotational speed. An equivalent mass–spring–damper system is investigated to find a closed-form expression describing this dependency. By normalizing the conventional imbalance diagnostic feature by the obtained dependency, a diagnostic feature is proposed. By conducting comprehensive experimental trials with a wind turbine with a permissible imbalance, it is justified that the proposed simplified version of imbalance diagnostic feature is speed-invariant.

## 1. Introduction

A rotor imbalance is one of the most frequent faults within wind turbines, and it can be corrected if diagnosed early [1]. However, a wind turbine imbalance may lead to a catastrophic failure if not diagnosed and treated early [1]. Aside from the direct effect of a rotor imbalance on increasing the stress level in turbine components, this fault has an adverse effect on the turbine drivetrain, leading to unwanted quality loss within the generated power [2].

Wind turbine fault diagnosis has been widely addressed in the literature to date. The available diagnostic methods in the literature are generally divided into two classes of (I) model-based [3,4,5] and (II) data-driven approaches [6,7,8,9], each of which has its own advantages and limitations. Model-based techniques require strong physical backgrounds, which can be challenging to obtain without sufficient knowledge of the system specifications. On the other hand, data-driven approaches need sufficient training data covering all faulty conditions, which are expensive to obtain. Here, the most pioneering works dealing with imbalance fault diagnosis are reviewed.

Imbalance fault diagnosis has been reported in many works to date. Referring to the fact that the additional centrifugal force coming from the mass imbalance rotates with the rotational speed of the machine, this fault is reflected in the fundamental harmonic intensity of the vibration signal [10,11,12,13,14]. Besides the vibration-based diagnosis, an imbalance fault can also be diagnosed through monitoring the stator current signal [15,16,17]. Because the centrifugal excitation coming from the imbalance fault makes the airgap unequal during the rotation of the induction machine rotor. So, the fault is reflected at the rotational speed sidebands around the supply frequency [15,16,17]. Instead of monitoring the intensity of certain frequencies, Ewert [18] showed that bispectral analysis can also be adopted for mass imbalance diagnosis. It was demonstrated that despite spectral analysis, the shortage of the registration time of the analyzed signal does not have any negative impact on bispectral diagnosis. Processing the vibration and current signals, it was found that bispectral analysis is more sensitive to the imbalance fault in the case of vibration-based diagnosis. These references dealt with systems operating at a single constant rotational speed.

Aside from single-speed systems, some rotating machines operate at multiple constant rotational speeds. Rafaq et al. [19] monitored the vibration signal collected from a permanent magnet synchronous motor operating at multiple constant rotational speeds. Regarding the fact that all the load-related faults like mass imbalance, rotor eccentricity and asymmetric demagnetization are reflected in the fundamental harmonic intensity of the vibration signal, a signature for distinguishing between the mass imbalance fault and others was proposed. It was shown that the strong dependency between the rotational speed and the intensity of the vibration signal fundamental rotation harmonic is a signature of the mass imbalance fault [19]. This paper did not provide diagnostic technology and just referred to this important feature of mass imbalance fault. Puerto-Santana et al. [20] investigated the imbalance fault in a general rotor-disc-bearing system. Regarding the dependency between the rotational speed and the intensity of the vibrational fundamental harmonic, their technology was based on calibrating the diagnostic system at each level of rotational speed. Their study did not cover nonstationary conditions and investigated systems operating at multiple constant speeds.

Focusing on wind turbines, Ramlau and Niebsch [21] simulated the dynamical behavior of these systems under imbalance fault. Solving the inverse problem that linked imbalances and the resulting nacelle vibrations, like other references reviewed above, it was also reported that the imbalance fault is reflected in the fundamental rotation harmonic intensities. The rotational speed was kept constant in this study to generate the results. This is not compatible with the operational conditions of wind turbines, as their rotational speed is continuously varying due to wind speed fluctuations.

Ewert et al. [22] employed the short-time Fourier transform together with the artificial neural network to diagnose the imbalance fault in a servo-drive dual rotor system. They also reported that the imbalance fault is reflected in the fundamental rotation harmonic intensity. Despite nonstationary signal processing, this paper provided results for a system operating at only a single constant speed.

Despite systems operating at single or multiple constant speeds, reviewed above, there exists a limited number of works dealing with imbalance fault diagnosis at variable rotational speed conditions. Li et al. [23] established the wind turbine model and simulated rotational inertia, rotational speed and generator output power signals using G.H. Bladed, version 4.2, software. Adopting these simulated signals, the aerodynamic torque signal was obtained and reconstructed through the order tracking method. The amplitudes of the first and the third rotational harmonics of the aerodynamic torque signal were obtained. As the wind turbine had three rotating blades, it was observed that the amplitude of the first rotational harmonic takes very small values in comparison to those of the third ones for systems working under healthy conditions. However, for systems with an imbalance fault, the amplitude of the fundamental rotational harmonic started increasing. It was reported, that the imbalance fault occurs if the ratio between the first and the third harmonic amplitudes takes values greater than one-third.

According to the results reported by Li et al. [23], the dependency between the intensity of the third rotational harmonic and the rotational speed is very weak in comparison to the speed dependency of the fundamental rotation harmonic intensity. Although their paper did not investigate the speed dependency of the ratio between the first and the third harmonic amplitudes as the diagnostic feature, one can conclude that their proposed fault indicator should not be speed-invariant. Hence, like the amplitude of the fundamental vibration harmonic, this feature still may depend on the rotational speed. Consequently, its magnitude can vary by changing either the fault severity or the rotational speed. This means that the diagnostic system cannot judge the source of change by monitoring the magnitude of this feature.

Adopting the order tracking method, which can be implemented either based on the speed data from a tachometer sensor [24,25] or without these data [26,27,28,29,30], Wu et al. [31] adopted a combination of the short-time Fourier transform and the seam carving algorithm to propose a tacholess order tracking method. Extracting the instantaneous frequency of the rotor and instantaneous phase under variable-speed conditions, the original nonstationary vibrational signal was transformed from the time domain to the angle domain and then to the order domain. In doing so, in comparison to the short-time Fourier transform, the fundamental rotation harmonic intensity as the fault indicator was more accurately extracted. However, the dependency between this intensity and the rotational speed was also not addressed in this study.

Xu et al. [32] diagnosed the imbalance fault in a 750 kW wind turbine using the complex Morlet wavelet transform. In processing the vibrational signal collected from the surface of the drive train, the amplitudes of the first three harmonics were obtained. Selecting the sum of the first and the second rotational harmonic intensities divided by the third harmonic magnitude of the vibrational signal as the diagnostic feature, it was shown that this new feature is sensitive to the amount of imbalance. Since the dependency of the fundamental rotation harmonic intensity on the rotational speed is much stronger than the intensities of other harmonics (e.g., the second or the third ones), the proposed feature is expected to be still speed dependent. However, this issue was not investigated in this study.

Based on the literature reviewed above, it is seen that the fundamental rotation harmonic intensity should be monitored to diagnose imbalance fault based on processing the vibration signal. However, besides the difficulties involved in accurately extracting the harmonic intensities in nonstationary conditions, the strong dependency between the fundamental rotation harmonic intensity and the rotational speed, in turn, makes the diagnosis challenging in variable-speed conditions. Because diagnostic systems working based on the fundamental rotation harmonic intensity cannot distinguish between the influence of the imbalance amount and the rotational speed on changing the level of the diagnostic feature, the feature can simply increase with an increase in the rotational speed without any change in the fault severity.

To the best of the authors’ knowledge, the dependency between the imbalance fault indicator and the rotational speed has not been investigated in the previous studies. Therefore, this work aims to investigate this dependency in wind turbines as variable-speed rotating machinery and to develop a novel speed-invariant feature for imbalance fault diagnosis. Therefore, the dynamics of an imbalanced wind turbine rotor as an equivalent mass–spring–damper model under centrifugal excitation is assessed to obtain a closed-form expression describing the dependency between the rotational speed and the vibrational fundamental rotation harmonic intensity. Adopting this closed-form expression, the fundamental rotation harmonic intensity is normalized to have a speed-invariant imbalance diagnostic feature. To verify the speed independency of the proposed feature, a time–frequency technique, the short-time chirp Fourier transform [33], is employed to process the experimental vibration data, collected from a 2.3 MW wind turbine with a permissible imbalance. Processing the nonstationary vibration data shows that, despite the different behavior of the fundamental rotation harmonic intensities in low- and high-speed regimes, the proposed feature is speed-invariant and behaves stably over the whole operational range.

The novelty of the present work is to propose for the first time in the worldwide term an imbalance diagnostic feature that is independent from the shaft rotational speed.

In view of the above-mentioned novelty, the objectives of the present study are as follows:

- To investigate the nonstationary dynamics of a wind turbine rotor via an equivalent mass–spring–damper system under centrifugal excitation.
- To find a closed-form expression describing the dependency between the rotational speed and the fundamental rotation harmonic intensity.
- To propose a speed-invariant diagnostic feature for imbalance fault diagnosis in wind turbines via simplifying the closed-form dependency for low-speed systems.
- To adopt the short-time chirp Fourier transform and process the experimental vibration data collected from a 2.3 MW wind turbine with a permissible imbalance.
- To obtain the local interference level and obtain the residuals describing the net feature values.
- To normalize the residuals by the local interference levels.
- To extract the conventional and the proposed diagnostic features and to investigate their dependencies on the rotational speed.

The organization of the present paper is as follows. Section 2 investigates the dynamics of an equivalent mass–spring–damper model associated with a wind turbine rotor under centrifugal excitation and provides a closed-form expression describing the dependency between the rotational speed and the fundamental rotation harmonic intensity. The details of the present diagnosis technology, including the short-time chirp Fourier transform as the processing tool, the diagnostic feature and its extraction procedure, are presented in the rest of this section. The details of the data-capturing system, installed at the nacelle compartment of the wind turbine, are also provided in this section. The speed independency of the proposed fault indicator is discussed in Section 3. The conclusions are summarized in Section 4.

## 2. Theoretical Background, Diagnostic Feature Proposition and Experimental Setup

### 2.1. Theoretical Background

**Figure 1:**
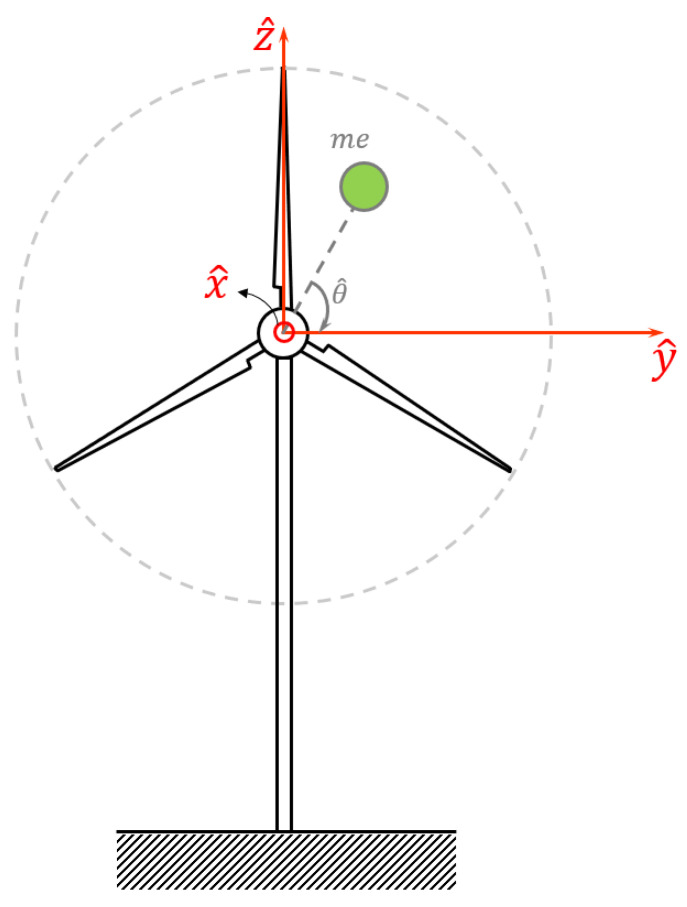
Schematic of a wind turbine with an imbalance mass.

Figure 1 shows a schematic of a wind turbine with an arbitrary level of imbalance, in which the $\hat{x}$, $\hat{y}$ and $\hat{z}$ directions describe the axis along the turbine’s main shaft, the horizontal axis perpendicular to the turbine’s main shaft and the vertical axis, respectively. In addition, m denotes the resultant imbalance mass placed in the virtual location e [34]. In this case, the centrifugal force $F_{C}$ applied to the main bearing of the turbine is [34]

(1) $$F_{C}=me{\hat{\Omega }}^{2}\;,$$

where $\hat{\Omega }$ stands for the variable rotational speed of the rotor, and the product of m×e is known as the amount of imbalance reported in gr.mm [34].

As the imbalance excitation occurs in the plane of blade rotation, it can be reflected over either the $\hat{y}$ or $\hat{z}$ axis. Given the lower stiffness of the wind turbine tower in the horizontal axis in comparison to that of the vertical direction, the occurrence of horizontal oscillations is more expected. Therefore, without reducing the generality of the model, we shall focus on the $\hat{y}$ direction and provide the governing equations for this direction. The only difference between these two directions is related to the trigonometric function describing the reflection of the centrifugal force on each direction. This trigonometric function, which is cosine for the $\hat{y}$ direction and sine for the $\hat{z}$ direction, does not affect the intensity of the fundamental harmonic of the vibrational signal. However, the validity of taking the $\hat{y}$ direction as the suitable direction for imbalance diagnosis is assessed in the next section.

As the instantaneous rotational speed $\hat{\Omega }$ is the first derivative of the rotational angle θ with respect to the time $\hat{t}$, one can write $\theta \left(\hat{t}\right)=\int_{0}^{\hat{t}}\hat{\Omega }\left(\hat{\tau }\right)d\hat{\tau }$. Therefore, focusing on the $\hat{y}$ direction, the horizontal component of the centrifugal excitation takes the form of

(2) $$F_{Cy}=me{\hat{\Omega }}^{2}cos\left(\int_{0}^{\hat{t}}\hat{\Omega }\left(\hat{\tau }\right)d\hat{\tau }\right)\;.$$

Modelling the wind turbine rotor by an equivalent mass–spring–damper system, the governing equation of motion takes the form of [35]

(3) $$\frac{d^{2}\hat{y}}{d{\hat{t}}^{2}}+2\xi \omega_{n}\frac{d\hat{y}}{d\hat{t}}+\omega_{n}^{2}\hat{y}=\frac{me{\hat{\Omega }}^{2}}{M}cos\left(\int_{0}^{\hat{t}}\hat{\Omega }\left(\hat{\tau }\right)d\hat{\tau }\right),$$

where ξ, $\omega_{n}$ and *M* are the equivalent damping ratio of the system, its natural frequency and its equivalent mass at the location of the accelerometer.

It is known that any continuous smooth function can be approximated by piecewise linear functions [36,37]. Thus, it is assumed that the whole time-domain signal is subdivided into time segments, in which the rotational speed is linearly approximated. By doing so, one can write

(4) $$\hat{\Omega }\left(\hat{t}\right)={\hat{\Omega }}_{0}+{\hat{\alpha }}_{0}\hat{t}\;For\;T_{i}\leq \hat{t}<T_{i+1}\;,$$

where ${\hat{\Omega }}_{0}$ and ${\hat{\alpha }}_{0}$ are the initial constant rotational speed and the chirp rate (i.e., frequency speed) at the *i*th (*i* = 1, 2, 3, …) time segment starting from $T_{i}$ and ending with $T_{i+1}.$

For convenience, the following dimensionless variables are introduced:

(5) $$y=\frac{\hat{y}}{e},\;t=\omega_{n}\hat{t},\;\Omega_{0}=\frac{{\hat{\Omega }}_{0}}{\omega_{n}},\;\alpha_{0}=\frac{{\hat{\alpha }}_{0}}{\omega_{n}^{2}},\;\lambda =\frac{m}{M}\;,$$

where y, t, $\Omega_{0}$, $\alpha_{0}$ and *λ*denote the normalized forms of the displacement, time, rotational speed, chirp rate and imbalance mass, respectively.

Upon substituting the dimensionless quantities given in Equation (5) into Equation (3), the normalized equation governing the motion of the system at the *i*^th^ time segment takes the form of

(6) $$\frac{d^{2}y}{dt^{2}}+2\xi \frac{dy}{dt}+y=\lambda {\left({\alpha_{0}t+\Omega }_{0}\right)}^{2}cos\left(\frac{1}{2}\alpha_{0}t^{2}+\Omega_{0}t+\theta_{0}\right)\;,$$

where $\theta_{0}$ is the angle of imbalance at that time segment [34].

Equation (6) is a second-order differential equation with initial conditions. This equation can be solved either analytically [38] or numerically using the fourth-order Runge–Kutta method [37]. The analytical solution is complicated and the numerical solution can provide acceptable accuracy for cases far away from the resonance area [35], in which an imbalance diagnosis should be performed to avoid signature of other faults [11]. Therefore, a numerical solution is taken in this paper.

Given the fact that the vibration signal, measured by accelerometers, describes the acceleration of the system versus time, one should employ Equation (6) to obtain the acceleration (i.e., $d^{2}y/dt^{2}$) if the displacement and the velocity are known as the outputs of the fourth-order Runge–Kutta method [37,39]. The fourth-order Runge–Kutta method is implemented via the MATLAB, version R2024a, command ODE45 in this study. Having the acceleration of the system versus the time, the amplitude of the acceleration is obtained using the MATLAB command envelope.

To obtain the dependency between the rotational speed and the fundamental rotation harmonic intensity, one should investigate the variation of the amplitude of the acceleration signal versus the rotational speed. In view of the experimental data, whose details will be discussed later, it is seen that the maximum chirp rate recorded for the present wind turbine does not exceed 0.01 Hz/s. Employing the in-house MATLAB finite element code, the lowest resonance frequency of the thick steel shaft of the present wind turbine is obtained as $\omega_{1}=103.05\;Hz$. Hence, the normalized chirp rate associated with the present wind turbine is at most equivalent to $\alpha_{0}=\frac{{\hat{\alpha }}_{0}}{\omega_{n}^{2}}≅\frac{0.01}{100^{2}}=10^{-6}$. To cover the worst-case scenario, which may occur in the run-up or gust [2] cases associated with most large wind turbines, it is assumed that the value of the normalized chirp rate is within the range from $10^{-4}$ to $10^{-2}$. To provide the results, reasonable damping ratios of 0.05, 0.1, 0.2 and 0.4 are also selected.

**Figure 2:**
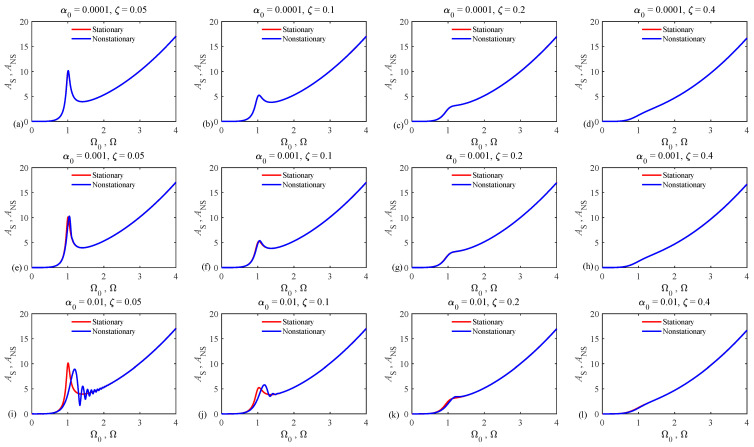
Variation of the normalized amplitude of acceleration versus the normalized instantaneous frequency in both stationary and nonstationary conditions. The chirp rate and damping coefficient are set to (**a**) $\alpha_{0}=0.0001,\;\zeta =0.05$, (**b**) $\alpha_{0}=0.0001,\;\zeta =0.1$, (**c**) $\alpha_{0}=0.0001,\;\zeta =0.2$, (**d**) $\alpha_{0}=0.0001,\;\zeta =0.4$, (**e**) $\alpha_{0}=0.001,\;\zeta =0.05$, (**f**) $\alpha_{0}=0.001,\;\zeta =0.1$, (**g**) $\alpha_{0}=0.001,\;\zeta =0.2$, (**h**) $\alpha_{0}=0.001,\;\zeta =0.4$, (**i**) $\alpha_{0}=0.01,\;\zeta =0.05$, (**j**) $\alpha_{0}=0.01,\;\zeta =0.1$, (**k**) $\alpha_{0}=0.01,\;\zeta =0.2$, (**l**) $\alpha_{0}=0.01,\;\zeta =0.4$.

Figure 2 illustrates the variation of the acceleration amplitudes versus the normalized instantaneous rotational speed (i.e., $A_{NS}-\Omega$ curves) for the chirp rates and damping ratios mentioned above. In this figure, the rotational speed Ω is normalized with respect to the system resonance frequency and amplitude $A_{NS}$ is given by

(7) $$A_{NS}=\frac{\ddot{Y}}{\lambda }\;,$$

where $\ddot{Y}$ denotes the amplitude of dimensionless acceleration.

In addition, this figure provides a comparison between stationary and nonstationary results. Neglecting the nonstationary conditions (i.e., setting $\alpha_{0}=0$ in Equation (6)), the stationary amplitudes of acceleration can be obtained as [35]

(8) $$A_{S}=\frac{\Omega_{0}^{4}}{\sqrt{{\left(1-\Omega_{0}^{2}\right)}^{2}+{\left(2\xi \Omega_{0}\right)}^{2}}}.$$

As Figure 2 demonstrates, the acceleration–frequency curves associated with the nonstationary conditions match those corresponding to the stationary cases for systems operating at small chirp rates, whose corresponding normalized values are lower than or equal to $10^{-2}$, which normally apply to most large wind turbines. The stationary and nonstationary curves differ from each other for systems with low damping ratios, and the normalized chirp rate of $\alpha_{0}=0.01$ (see Figure 2i,j). However, this mismatch occurs near the resonance zone, which is far away from the imbalance detection area, namely areas with normalized frequencies lower than 0.5 and higher than 2 [11,20,35]. Thus, the dependency between the normalized amplitude of acceleration and the rotational speed in the imbalance detection area for systems with a maximum normalized chirp rate lower than or equal to $\alpha_{0}=0.01$ is governed by Equation (8), which has a universal character.

Based on the dependency between the rotational speed and the fundamental rotation harmonic intensity, it is proposed here to normalize the fundamental rotation harmonic intensity in order to obtain a speed-invariant imbalance fault indicator. The proposed novel imbalance feature ($S_{N}$) takes the form of

(9) $$S_{N}=\frac{S}{C},$$

where *S* is the intensity of the fundamental rotation harmonic of the signal, and the normalizing coefficient *C* takes the form of

(10) $$C=\frac{\Omega^{4}}{\sqrt{{\left(1-\Omega^{2}\right)}^{2}+{\left(2\xi \Omega \right)}^{2}}}\;.$$

Taking into account the coincidence between amplitudes $A_{NS}$ and $A_{N}$ in areas which are far away from the resonance zone, Equation (10) is valid for rotating machinery operating in low chirp rate nonstationary conditions. Therefore, the instantaneous normalized rotational speeds can be employed in Equation (10), which has a universal character and is valid for any variable speed rotating machinery, including for large wind turbines, if their maximum normalized chirp rates do not exceed $\alpha_{0}=0.01$.

The fundamental harmonic intensities, as the conventional imbalance diagnostic feature, is a function of the amount of imbalance, the system rotational speed, the corresponding resonance frequency of the rotor and its equivalent damping ratio. Since phenomena like wind speed change, tower shadow, etc. do not affect rotor resonance frequency and its equivalent damping ratio, the proposed diagnostic feature is robust against these phenomena for all wind turbines, especially for large wind turbines, whose large inertia prevents them from experiencing high chirp rates.

Given the fact that the intensity *S* depends on both the amount of an imbalance and the rotational speed, and coefficient *C* describes its dependency on the rotational speed, feature $S_{N}$ will only be a function of the amount of imbalance.

According to Equation (10), aside from the rotational speed, the normalizing coefficient depends on the resonance frequency of the system and on its damping ratio. Determining an equivalent damping ratio is a challenging task [40]. To investigate this issue, variation of the normalizing coefficient *C* versus Ω and ξ is assessed. To do so, cases in which one can estimate the equivalent damping ratio of the system with an accuracy of 0.05 are considered.

The whole diagnosis region is divided into two areas: before and beyond the resonance zone (i.e., the areas with a normalized rotational speed lower than 0.5 and higher than 2). Table 1 considers three different values of Ω 0.15, 0.3 and 0.45 before the resonance area and three different values of Ω 2, 3 and 4 beyond the resonance area. In addition, three different ranges of 0.05≤ζ≤0.1, 0.15≤ζ≤0.2 and 0.3≤ζ≤0.35 for the damping coefficient are considered. Table 1 provides the maximum relative error (${RE}_{max}$) occurred due to using an averaged value for the damping ratio over each interval instead of its exact value, as follows:

(11) $${RE}_{max}=max\left(\frac{\left|C\left(\xi ,\Omega \right)-C\left(\bar{\xi },\Omega \right)\right|}{C\left(\xi ,\Omega \right)}\right)\times 100\%\;,$$

where $\bar{\xi }$ denotes the average damping ratio over each interval.

As Table 1 shows, the normalizing coefficient *C* is not as sensitive to the damping ratio as to the normalized frequency. Thus, given the universal character of the normalizing coefficient *C*, which is valid for all rotating machinery including large wind turbines when their normalized chirp rate does not exceed $\alpha_{0}=0.01$, achieving good accuracy for the resonance frequency, and consequently the normalized frequency, is sufficient to accurately obtain the dependency between the fundamental harmonic intensity and the rotational speed and there is no need for the exact damping ratio of the system.

For low-speed systems operating in the Ω≪1 region, the novel simplified version of the general feature $S_{N}$, given in Equation (9), is proposed below. In this case, one can approximate ${\left(1-\Omega^{2}\right)}^{2}≅1$, which results in the denominator of the normalizing coefficient, given in Equation (10), being simplified to $\sqrt{1+{\left(2\xi \Omega \right)}^{2}}$. The expression (10) can be simplified further if either the damping ratio of the system is less than 0.5 or the rotational speed of the system takes values sufficiently smaller than its resonance frequency, so that $4\xi^{2}\Omega^{2}\ll 1$. In these cases, one obtains

(12) $$C_{app}=\Omega^{4}\;,$$

where $C_{app}$ denotes the approximated version of the normalizing coefficient *C.*

**Figure 3:**
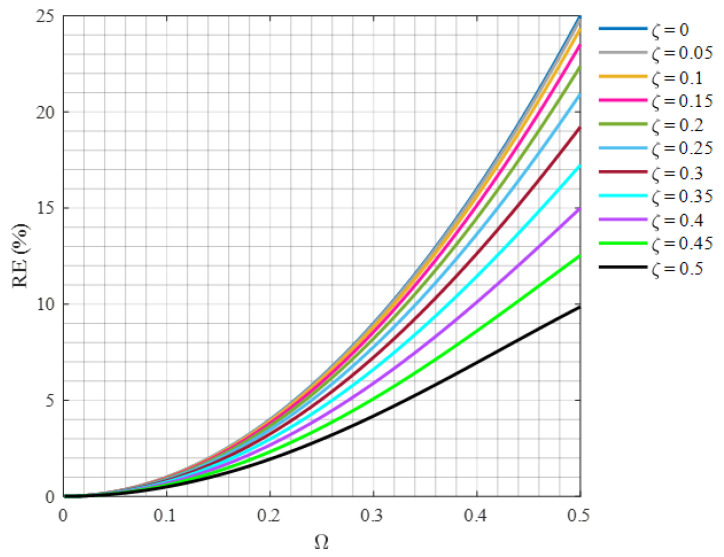
Relative error due to adopting normalization (12) versus the normalized rotational speed for different values of the damping ratio.

Equation (12) states that the normalizing coefficient depends on neither the resonance frequency of the system nor its equivalent damping ratio. To quantify the inaccuracy involved in using $C_{app}$ instead of *C*, Figure 3 provides the relative error (RE) associated with this approximation according to the following expression:

(13) $$RE\left(\xi ,\Omega \right)=\frac{\left|C-C_{app}\right|}{C}\times 100\%\;.$$

As Figure 3 indicates, adopting coefficient $C_{app}$ instead of coefficient *C* for systems with Ω≤0.2 will introduce a relative error of at most 4%. This relative error for the present turbine case, whose maximum rotational speed does not exceed 0.3 Hz, will be less than 0.1%. Therefore, the proposed normalization (12) provides an acceptable accuracy. The proposed imbalance diagnostic feature could be presented as

(14) $$S_{N,\;Simp}=S/C_{app}.$$

Taking into account the attractiveness of feature $S_{N,\;Simp}$, given in Equation (14) (i.e., only rotational speed data are needed for normalizing the fundamental rotation harmonic intensity), and its applicability for large wind turbines, this feature will be further investigated here.

### 2.2. Feature Extraction

Given the inaccuracy involved in the short-time Fourier transform [31], this study adopts the short-time chirp Fourier transform instead [33], which is effectively used for fault diagnosis under variable frequency conditions [41,42,43,44]. Considering $x\left(t\right)$ as a digital time signal (the vibrational signal in the present case), the short-time chirp Fourier transform, as the extension of the chirp Fourier transform [45], states [33]

(15) $$X\left(f,T\right)=\frac{1}{T}\int_{-\infty }^{\infty }h\left(t-T\right)x\left(t\right)exp\left[-j2\pi \left(\int_{0}^{t}f\left(\tau \right)d\tau \right)\right]dt\;,$$

where $h\left(t\right)$ is the time window, *f* is the instantaneous frequency in Hz from a tacho sensor and *T* is the window duration.

After taking the short-time chirp Fourier transform, the peak value, corresponding to the average rotational speed over each time window, is selected. To obtain the peak value associated with the imbalance fault, the level of the interference around the fundamental rotational harmonic should be subtracted. To do so, the average level of the local interference around the peak is given by [46,47]

(16) $$N_{m}=\frac{\sum_{i=1}^{n}N_{i}^{left}+\sum_{i=1}^{n}N_{i}^{right}}{2n}\;,$$

where $N_{i}^{left}$ denotes the intensity of the *i*th component from the peak on its left-hand side and $N_{i}^{right}$ is the intensity of the *i*th one on the right-hand side. Herein, leaving the nearest component to the peak at each side, the value of *n* is set to 3. Having the average local interference (i.e., $N_{m}$), the residual peak value takes the form of [46,47,48]

(17) $$R=\sqrt{P^{2}-N_{m}^{2}}\;,$$

where *P* denotes the peak value.

After extracting the residual peak values, they should be normalized by the average local interference level [39]. This refers to the conventional imbalance fault indicator given by

(18) $$S=\frac{R}{N_{m}}\;,$$

where $S_{N,\;Simp}$ is evaluated through dividing *S* by the average rotational speed to the power of four over each time window. Since *S* is dimensionless, the unit of $S_{N,\;Simp}$ is Hz^−4^ or s^4^.

### 2.3. Experimental Setup

This paper proposes a speed-invariant imbalance diagnostic feature for rotating systems, operating at variable speeds with low chirp rates based on a universal theoretical background. To justify the speed independency of the proposed diagnostic feature, the vibration data are collected from a 2.3 MW wind turbine with a permissible level of imbalance, lower than $5.94\times 10^{8}\;g\cdot mm$ [1,34], over a long period of turbine operation. This turbine is a horizontal-axis three-blade machine with a blade length of 40 m and a variable rotational speed which does not exceed 0.3 Hz.

**Figure 4:**
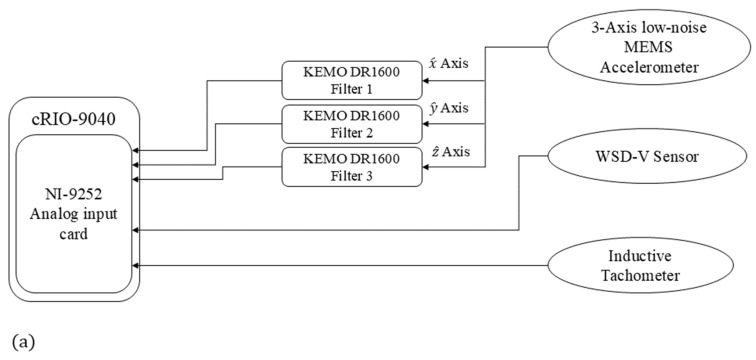

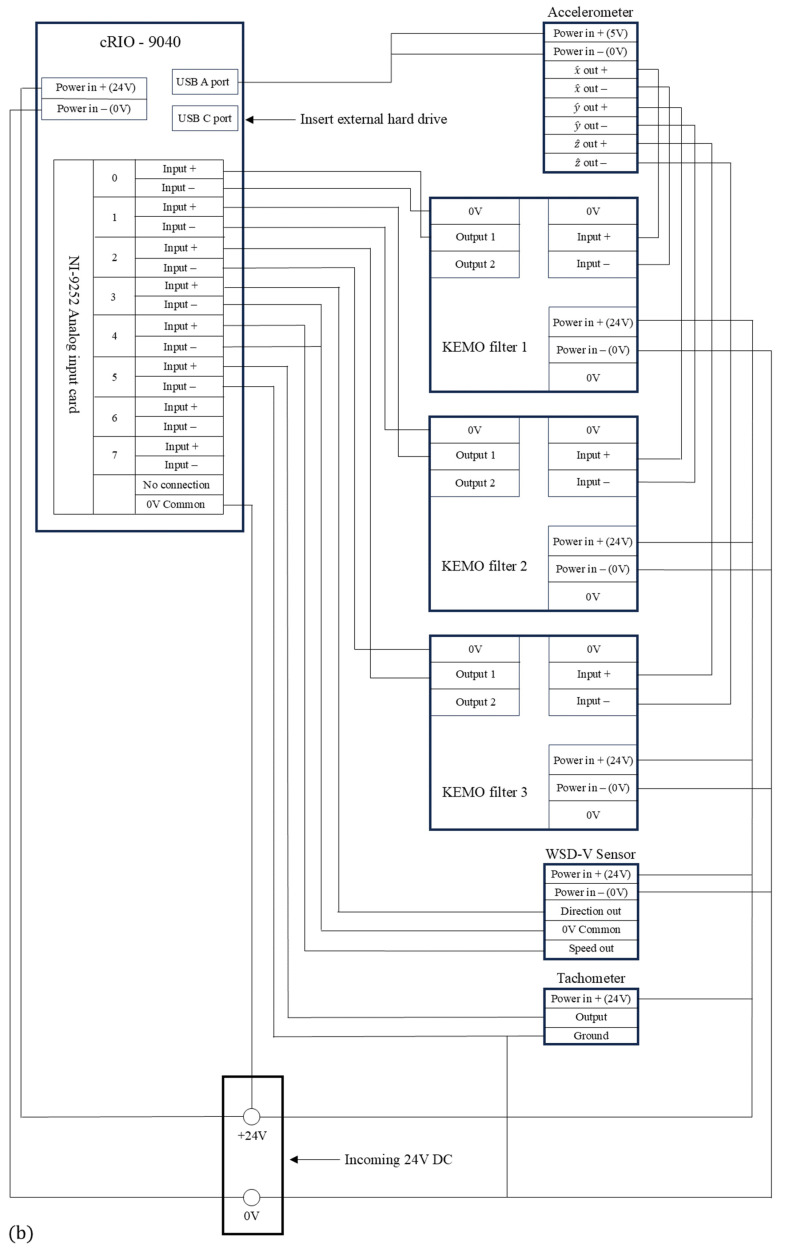
Data-capturing system (**a**) schematic, (**b**) device diagram.

To collect vibration data, a data-capturing system, whose schematic is provided in Figure 4a, is installed in the nacelle compartment of the wind turbine. This system includes (i) an accelerometer, (ii) KEMO filters, (iii) a tachometer, (iv) a wind speed and direction sensor, and (v) a web data acquisition system.

The accelerometers are installed on the bearing of the main shaft of the wind turbine so that its different axes match those presented in Figure 1. This location is selected to capture accelerations as close as possible to the turbine blades, in which the occurrence of an imbalance fault is most probable. Two MEMS accelerometers are employed: (a) ADXL354B (Manufactured by Analog Devices Inc. (Mixed-signal and digital signal processing ICs|Analog Devices), Sourced from the UK), (b) PCB 3743F112G (Manufactured by PCB Piezotronics Inc. (PCB Piezotronics|Measure vibration, pressure, force, and more), Sourced from the UK). The details of the specifications of these sensors are given in Table 2.

To amplify the output of the accelerometer and delimitate their frequency bandwidth, DR 1600 KEMO anti-aliasing (Manufactured by KEMO Ltd. (High performance High/Low Pass Signal Filter Solutions for industry since 1965), Sourced from the UK) filters with 3 W power input, −10 °C to 45 °C operating temperature range, bandwidth of 500 kHz and a total harmonic distortion lower than 0.003% are used. This filter is adjustable and provides a wide range of gains, including ×1, ×2, ×5, …, ×1000. The adopted gains of these filters are set to ×10. The cut-off frequencies are also set to 10 kHz.

To measure the rotational speed of the turbine’s main shaft, a 32 pulse per revolution LJ12A3-4-Z/BY inductive proximity sensor with 500 Hz response frequency, −25 °C to 55 °C operating temperature range and detection range of 4 mm is employed.

The wind speed is recorded through an MSL WSD-V sensor with the capability of measuring wind speeds higher than 0.5 m/s up to 50 m/s with an accuracy of 1.5 m/s and a resolution better than 0.5 m/s. The operational temperature range of this sensor for the head unit is −20 °C to 60 °C.

Since the outputs of all the sensors are analog, an eight-input NI-9252 analog input card with 24-bit resolution and a maximum sampling rate of 50 kS/s on the chassis of the cRIO-9040 system with a maximum input/output frequency of 20 MHz and −20 °C to 52 °C operating temperature range is employed. This web data acquisition card is set to simultaneously sample all signals with a rate of 25 kS/s.

As Figure 4b demonstrates, the MEMS accelerometers are fed via USB port A, available on the data acquisition system. The outputs of these sensors are connected to KEMO filters, fed by a 24 V input DC voltage. The outputs of the KEMO filters are connected to the analog input card on the system. The WSD-V and inductive proximity sensors are directly connected to the analog input card and fed by the same 24 V DC input voltage. The recorded digital signals are transferred and saved to an external hard drive via USB port C on the system.

## 3. Results and Discussion

The present vibration data are captured and saved within 60 min time portions from a 2.3 MW wind turbine, as a typical representative of wind farm turbines, over a sufficiently long period of operation, covering multiple wind speeds and multiple rotational frequencies over the whole operational frequency range. Taking a 50 s time window with 40% overlapping, features *S* and $S_{N,\;Simp}$ are extracted in all three directions. The total number of each feature (i.e., feature *S* or feature $S_{N,\;Simp}$ in each direction) is 8181, the rotational speeds are in the range of 0.17 to 0.27 Hz and the minimum and maximum wind speeds over the investigated period take the values of 4.7 m/s and 10.7 m/s, respectively. Since the aim of the experiment is to justify the speed independency of the proposed diagnostic feature, and the collected data cover all possible rotational speeds, the presented experimental justification will be sufficient for validating the presented theoretical background. Taking into account the universal theoretical character of the background, there is no need for adjusting the proposed diagnostic feature for other wind turbines.

**Figure 5:**
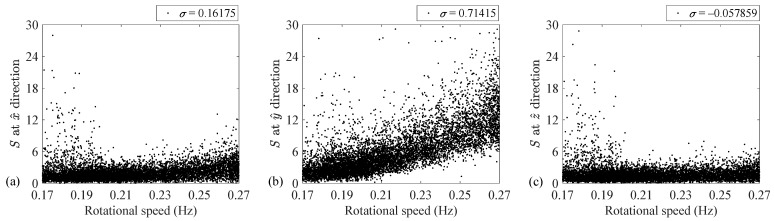
The conventional imbalance feature versus the rotational speed at (**a**) $\hat{x}$, (**b**) $\hat{y}$ and (**c**) $\hat{z}$ directions.

Every well-functioning piece of rotating machinery faces a permissible level of mass imbalance fault [34]. According to the available literature [19,34], dependency between the fundamental rotation harmonic intensity and the rotational speed indicates the existence of this fault. Figure 5 illustrates the variation of the conventional imbalance feature versus the rotational speed of the present wind turbine with permissible imbalance for all three directions. To quantify the dependency between these two parameters, the normalized cross covariance (i.e., *σ*) [49], corresponding to each direction, is also given in this figure. As Figure 5 demonstrates, this dependency is observable only for the $\hat{y}$ direction. This observation is consistent with an expectation because the $\hat{y}$ direction is placed within the plane of blade rotation, in which the centrifugal excitation, coming from the imbalance fault, has a reflection in this direction, and the stiffness of the turbine tower along this direction is less than the stiffness of the vertical direction (i.e., the $\hat{z}$ axis). Therefore, the $\hat{y}$ direction is the only candidate for imbalance diagnosis.

As Figure 5b illustrates, selecting the intensity of the fundamental harmonic at the $\hat{y}$ direction for imbalance diagnosis could produce a misleading interpretation because this conventional feature is dependent on the rotational speed. This feature can be increased either by increasing the rotational speed or by increasing an imbalance severity, or by increasing both parameters. Therefore, the occurrence of a wind turbine imbalance cannot be effectively detected via this conventional feature.

**Figure 6:**
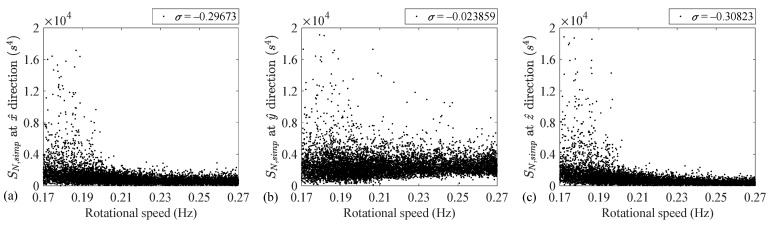
$S_{N,\;Simp}$ versus the rotational speed at (**a**) $\hat{x}$, (**b**) $\hat{y}$ and (**c**) $\hat{z}$ directions.

Given the fact that the conventional feature at the $\hat{y}$ direction reflects the imbalance fault, according to the above theoretical background, feature $S_{N,\;Simp}$ at this direction is speed-invariant. Illustrating the variation of feature $S_{N,\;Simp}$ versus the rotational speed, Figure 6 shows the independency of this feature from the rotational speed. To quantify the independence, the normalized cross covariances between this feature and the rotational speed, corresponding to each direction, are also given in this figure. As Figure 6 shows, despite the other two directions, feature $S_{N,\;Simp}$ at the $\hat{y}$ direction is independent from the rotational speed, i.e., the corresponding normalized cross covariance is very close to zero. For the other two directions (i.e., for the $\hat{x}$ and $\hat{z}$ directions) the proposed feature and the rotational speed are negatively correlated. This is due to the fact that the conventional imbalance features in these two directions are independent from the rotational speed, as illustrated by Figure 5. Hence, the division of the conventional feature by the rotational speed to the power of four leads to the negative cross-correlations for the $\hat{x}$ and $\hat{z}$ directions.

To investigate the speed independency of the proposed feature in more detail, the whole operational speed range is subdivided into two subranges: low- and high-speed subranges covering the intervals [0.17, 0.22] Hz and [0.22, 0.27] Hz, respectively. This subdivision includes 4955 features over the low-speed subrange and 3226 features over the high-speed subrange.

**Figure 7:**
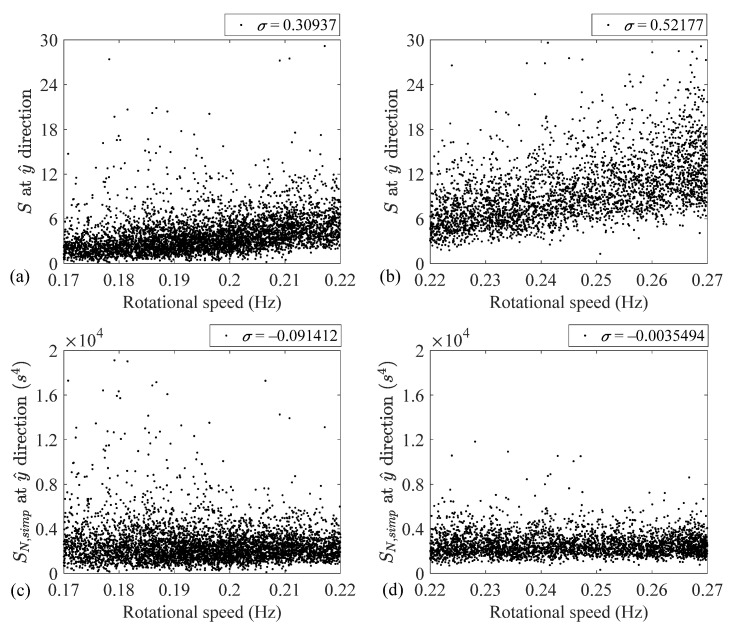
Speed dependency of *S* and $S_{N,\;Simp}$ over subranges: (**a**) *S* over the low-speed regime, (**b**) *S* over the high-speed regime, (**c**) $S_{N,\;Simp}$ over the low-speed regime and (**d**) $S_{N,\;Simp}$ over the high-speed regime.

To investigate the speed dependency of features *S* and $S_{N,\;Simp}$ at the $\hat{y}$ direction over low- and high-speed regimes, Figure 7 depicts the variations of these two features over the subranges and provides the corresponding normalized cross variance values. As this figure illustrates, the conventional feature speed dependencies over the low- and high-speed subranges are slightly different. This slight difference is confirmed by different normalized cross-correlation coefficients, i.e., 0.31 and 0.52, respectively.

However, as Figure 7 demonstrates, feature $S_{N,\;Simp}$ is speed independent over the low- and high-speed subranges. Despite the slightly different behavior of the conventional imbalance feature over the low- and high-speed subranges, the proposed feature behaves similarly over the whole operational range.

**Figure 8:**
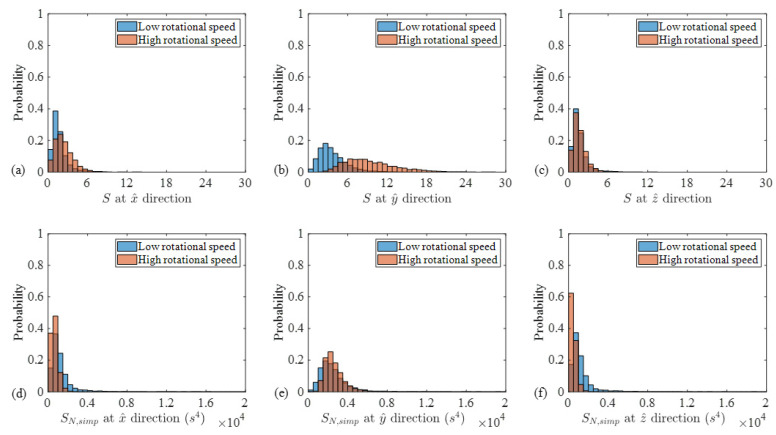
Histograms of *S* (**a**–**c**) and $S_{N,\;Simp}\;$ (**d**–**f**) associated with the low and high rotational speed regimes.

To complete the investigation, Figure 8 compares the probability density functions of the low- and high-speed cases for both *S* and $S_{N,\;Simp}$ in all three directions. The probabilities corresponding to the low-speed cases are colored blue and those associated with the high-speed cases are colored orange. In addition, the overlapping areas between the low- and high-speed cases are colored brown as the combination of blue and orange. To quantify the separation between the probabilities, Table 3 presents the values of the Fisher criterion (FC), associated with these cases. The FC is applied as [50,51,52]

(19) $$FC=\frac{{\left(m_{1}-m_{2}\right)}^{2}\;}{\mu_{1}^{2}+\mu_{2}^{2}}\;,$$

where $m_{i}$ and $\mu_{i}$ (i=1, 2) denote the mean value and the standard deviation of the *i*th probability density function, respectively.

As Figure 8 and Table 3 demonstrate, the separations between the low- and high-speed regimes agree with those observed previously. That is, the conventional imbalance fault indicator separations are almost zero except for the $\hat{y}$ axis. In addition, the FC for $S_{N,\;Simp}$, associated with the $\hat{y}$ axis, is also equal to zero.

Thus, it is seen that the experimental observations associated with the 2.3 MW wind turbine, as a variable-speed machine, verify the theory and emphasize that the feature $S_{N,\;Simp}$ is speed-invariant. Therefore, adopting the proposed feature for an imbalance diagnosis is recommended.

## 4. Conclusions

Based on the investigation of the dependency between the intensity of the fundamental harmonic of the vibration signal and the rotational speed, performed in this paper, the novel speed-invariant imbalance fault indicator and its novel simplified version, which is applicable for large wind turbines, are proposed for the first time in worldwide terms.

Assessing the response of an equivalent mass–spring–damper system under nonstationary centrifugal excitation, the dependency between the amplitude of the fundamental harmonic of the acceleration signal and the rotational speed is obtained. Analytical assessment shows that aside from the rotational speed, this dependency is a function of the resonance frequency and the equivalent damping ratio of the system. However, it is observed that for systems whose rotational speed is lower than 20% of their resonance frequency, the fundamental rotation harmonic intensity is a function of the rotational speed in power four.

The theory is verified by the experiment, in which the nonstationary vibration data from the main bearing of a variable-speed 2.3 MW wind turbine with a permissible imbalance are processed. Adopting the short-time chirp Fourier transform, the intensity of the fundamental vibration harmonic and the average level of the local interference around this harmonic are extracted.

The experimental results indicate that the normalized cross covariance between the conventional imbalance diagnostic feature in the $\hat{y}$ direction and the rotational speed is 0.71. This covariance is −0.02 for the case of the proposed feature $S_{N,\;Simp}$. Thus, the speed dependency of the conventional imbalance feature will be removed if it is being normalized with respect to the rotational speed in power four.

It is also observed that despite the different behavior of the conventional imbalance fault indicator over the low- and high-speed subranges, $S_{N,\;Simp}$ behaves similarly over the whole operational range. That is, the normalized cross covariances between this feature and the rotational speed are −0.09 and −0.003 over the low- and high-speed regimes, respectively.

Based on the performed investigations, as well as the similarity between the probabilities of $S_{N,\;Simp}$ over the low- and high-speed regimes, it is found that $S_{N,\;Simp}$ is speed-invariant. Thus, this feature is recommended for imbalance diagnosis for large wind turbines with low chirp rates.

This study is most suitable for imbalance diagnosis for variable speed machines with low chirp rates. The proposed diagnostic features present a novel conceptualization and will make an essential impact on an imbalance diagnosis for variable speed machines with low chirp rates, utilized in multiple industrial sectors.

This work presents a novel diagnostic feature to overcome the speed dependency of the conventional imbalance fault diagnostic feature. It can potentially be continued as follows:

- Investigate variable speed imbalance diagnosis in rotating machinery while simultaneously contracting a shaft misalignment.
- Investigate variable speed imbalance diagnosis in rotating machinery while simultaneously contracting blade fatigue cracks.

## Figures and Tables

**Table 1:** The maximum relative error (%) due to the use of the average damping ratio instead of its exact value within the given ranges.

	Ω=0.15	Ω=0.3	Ω=0.45	Ω=2	Ω=3	Ω=4
0.05≤ζ≤0.1	0.02	0.09	0.27	0.38	0.12	0.06
0.15≤ζ≤0.2	0.04	0.2	0.57	0.79	0.26	0.13
0.3≤ζ≤0.35	0.08	0.35	0.94	1.26	0.45	0.23

**Table 2:** Specifications of the accelerometers utilized.

	ADXL354B	PCB 3743F112G
Type	3-axis MEMS sensor	3-axis MEMS sensor
Sensitivity	200 mV/g	1350 mV/g
Measurement range	±2 g	±4 g
Operational frequency range	0–250 Hz	0–1.5 kHz
Resonance frequency	2.4 kHz	1.2 kHz
Typical nonlinearity	0.3%	0.1%
Typical transverse sensitivity	1%	1%
Operating temperature range	−40 °C to +125 °C	−54 °C to +121 °C
Temperature sensitivity change within the operational range	±0.01%/°C	±1%/°C

**Table 3:** Fisher criterion associated with the probabilities of *S* and $S_{N,\;Simp}$ for all three directions.

	*S*	$S_{N,Simp}$
$\hat{x}$ direction	0.05	0.13
$\hat{y}$ direction	1.28	0.000
$\hat{z}$ direction	0.002	0.15

## Data Availability

Data are unavailable due to privacy restrictions.
